# β-lactam allergy: clinical implications and costs

**DOI:** 10.1186/1476-7961-11-2

**Published:** 2013-11-27

**Authors:** Giovanni Satta, Victoria Hill, Marisa Lanzman, Indran Balakrishnan

**Affiliations:** 1 Department of Medical Microbiology, Royal Free London NHS Foundation Trust, London, UK; 2 Department of Pharmacy, Royal Free London NHS Foundation Trust, London, UK

**Keywords:** Penicillin, β-lactam, Allergy, Cost

## Abstract

**Background:**

β-lactam allergy is the most commonly reported medication allergy and it remains a key issue in antibiotic prescribing. A detailed and accurate history taking play a key role in preventing potentially serious clinical incidents and it may contribute in reducing costs.

**Methods:**

Data were collected for patients with a documented penicillin allergy on their drug chart during a six month period. Sources included the inpatient drug charts and medical notes. Adherence to hospital guidelines was audited and costs of treatments were calculated.

**Results:**

94 patients with a history of penicillin allergy were included. Compliance with the hospital antibiotic policy was 81% and 52% of cases had a description of the reaction documented. The mean additional cost per patient was £89.29 (excluding VAT).

**Conclusions:**

It is important to maintain a high level of vigilance and constantly educate all healthcare professionals involved in prescribing and dispensing antibiotics in order to avoid the unnecessary use of non-penicillin-based antibiotics and associated cost implication.

## Background

β-lactam allergy is the most commonly reported medication allergy and it is self-reported by at least 10% of patients. However, 90% of these individuals are found not to be allergic and are able to tolerate penicillins [1]. In many cases, β-lactam exposure might not actually have occurred, or patients may have had non-immunologic adverse events such as vomiting, diarrhea, or nonspecific rash, other non-toxic effects or contemporaneous side effects inappropriately attributed to the drug. These patients actually tolerate all β-lactams [2]. Inappropriate labeling of a patient as “penicillin allergic” can have a major impact on patient safety, the use of restricted antibiotics and the hospital budget.

As advised by BSACI (British Society of Allergy and Clinical Immunology) guidelines on drug allergy [3] every patient should be questioned about his/her allergy status prior to any drug prescription and results should be clearly recorded in the medical notes and drug chart. In addition, national and local antibiotic policies frequently give an alternative antibiotic choice in case penicillin cannot be used.

However, despite the presence of national and local guidance, compliance remains low. A recent Canadian study of inpatients on intensive care, coronary care or medicine wards found that the nature of the penicillin allergy was described in only 30% of cases [4]. This value matches with compliances of 33% in an outpatients setting of family medicine clinics [5] and 34% in inpatient charts [6].

As the options for the treatment of infections become more limited and increasingly costly due to the emergence of highly resistant organisms, it is important that we make the best use of the antibiotics available. In the hospital setting we are familiar with the use of evidence based guidelines to aid the appropriate prescribing of antibiotics, but quite often we fail to investigate the nature of antibiotic allergies which may preclude the use of cheap, effective agents. Antibiotics that are used more frequently and over a longer period of time have a higher prevalence of allergy. Additional risk factors include increasing age and female sex [7,8].

Based on this current lack of adherence and the need of a greater antibiotics stewardship, we decided to carry out an audit on the compliance with national and local guidelines, to better understand the problem and to tackle the main areas requiring improvements.

## Methods

We performed an audit to challenge history taking, documentation and compliance with hospital guidelines as regards β-lactam allergy. Data were collected on patients with a reported β-lactam allergy and treated with antibiotics in a tertiary university hospital (Royal Free London) during a 6 months period.

We evaluated compliance with the following standards: allergy history taken, documentation in the drug chart, more detailed documentation clarifying the nature of the allergy written in the medical notes and prescription made according to current antibiotic guidelines. Impact on clinical management and costs were also evaluated. Data were collected by a microbiologist/pharmacist using a predefined audit proforma.

## Results and discussion

We collected information on 94 patients who gave a history of penicillin allergy over a period of 6 months. The median age was 65 years (19–95) with a 1:2 male/female ratio. All medical and surgical specialties were represented. Chest infection was the commonest reason for the use of antibiotics (26%), followed by skin and soft tissue (18%), urinary tract and abdominal infections (both 16%) and sepsis of unknown origin (5%).

There was 100% compliance in recording penicillin allergy in the drug chart and in the medical notes and 81% of prescriptions were compliant with hospital antibiotic policy and/or were approved by Microbiology. However, in 19% of cases, no allergy history was recorded in the electronic notes (Cerner) and in only 52% of cases was the penicillin allergy better specified explaining the type of reaction. In this last group, rash was the commonest drug reaction noted (52%), followed by anaphylaxis (17%), other allergic symptoms (10%), gastrointestinal symptoms (10%) and unknown (5%). The term “intolerance” was written in an additional 6% of the notes but the clinical significance of this term was not explained and it was therefore unhelpful in understanding the type of reaction. Interestingly, only one patient out of 94 was referred for an allergology review and had a skin test performed.

With regard to patient safety, in three cases a penicillin-based antibiotic was wrongly prescribed and these constituted serious clinical incidents. In another two cases, the clinical teams discussed the nature of penicillin allergy with patient, family or GP and decided to use a penicillin-based antibiotic as the reaction was considered to be non-significant. In all cases, no allergic reactions were recorded. The history of β-lactam allergy did not have any impact on the choice of antibiotics in 30% of cases – the patients were commenced on non-penicillin-based antibiotics for other reasons, predominantly multi-resistant pathogens.

As regards costs, the total acquisition cost (excluding VAT) of antibiotics for the penicillin allergic group was £17,849 compared with £9,831 for equivalent treatment in non-allergy (based on hospital guidelines). This constitutes a mean extra cost per patient of £89.29. The commonest non-penicillin based alternative that was prescribed was a fluoroquinolone (30%), followed by a carbapenem (17%), a macrolide (14%), a glycopeptide (10%), an aminoglycoside (9%) and various combinations of these or other antibiotics.

Overall, the documentation of β-lactam allergy and compliance with guidelines were good, although the data were likely biased by the selection process. As regards clarification of the nature of the allergy, there is still scope for considerable improvement, even if our result (52%) is much higher than the reports previously described in the literature. Female sex and older age were confirmed as risk factors. Also β-lactam allergy still remains a key issue in antibiotic prescribing with potential clinical incidents. It is important to distinguish non-allergic reaction from true allergy (and the type of reaction), as this will have an important impact on the antibiotic choice. Although a myth persists that approximately 10% of patients with a history of penicillin allergy will have an allergic reaction if given a cephalosporin, a recent review showed that the overall cross-reactivity rate is approximately 1% (in particular when using first and second generations) [9]. In addition, it appears to be little/no potential cross reactivity between meropenem and penicillins even in patients with a definite history of anaphylactic reactions to penicillins [10].

There are clear cost-implications associated with β-lactam allergy. One previous paper has evaluated this in a community setting with calculated extra costs of $10.53 (~ £7) per patient [5] and three in the hospital setting with average additional costs of Can$91.12 (~£54) [4], €159 (~ £136) [11] and US$212.10 (~£132) [12]. The significantly higher hospital costs were attributed to increased expenditure on intravenous antibiotics instead of oral formulations. A history of penicillin allergy has also been associated with an increased length of stay and total cost of admission [13]. These cost-implications might be hidden by the rising prevalence of bacterial resistance and consequent use of non-penicillin-based alternatives. It should also be considered that a comparison of acquisition costs alone is unlikely to accurately reflect the total costs incurred when using treatments with significant toxicities or reduced efficacy [14].

Considering all these implications, we could suggest that all patients with a query penicillin allergy should be referred to a specialist for skin test and/or further tests. The main limitations against this would be the inability of services to cope with the increased demand, the possible non cost-effectiveness or simply general practitioners and hospital teams too busy (and not used to) to organize an additional referral for a non urgent problem. The main indications for skin testing should be the lack of an alternative antibiotic option and patients with recurrent infections and multiple allergies (thus with a limited choice of antibiotics). Education and training remain key issues.

## Conclusions

β-lactam allergy still remains a key issue in antibiotic prescribing. In order to protect patients, rationalize antibiotic prescribing and reduce costs, it is important to maintain a high level of vigilance and constantly educate all healthcare professionals. The need for detailed and accurate history taking, documentation, eventual referral to an allergy specialist, as well as an increased awareness and knowledge of possible alternative antibiotics, are the main areas where education and training are still required.

## Competing interests

The authors declare that they have no competing interests.

## Authors’ contribution

GS designed the study, collected the data and wrote the draft paper. ML, VH and IB helped with the design of the study, data collection and they revised the draft paper. GS and ML calculated the costs. All authors approved the final version.
